# Development of a novel diagnostic system for a telepsychiatric application: a pilot validation study

**DOI:** 10.1186/1756-0500-7-508

**Published:** 2014-08-09

**Authors:** Savita Malhotra, Subho Chakrabarti, Ruchita Shah, Aarzoo Gupta, Anurati Mehta, B Nithya, Vineet Kumar, Minali Sharma

**Affiliations:** Department of Psychiatry, Postgraduate Institute of Medical Education and Research (PGIMER), Chandigarh, 160012 India

**Keywords:** Telepsychiatry application, Adult psychiatric disorders, Diagnostic accuracy, Feasibility

## Abstract

**Background:**

A net-based, decision support system for diagnostic assessment and management of psychiatric disorders, developed as part of a telepsychiatry service, which aims to deliver mental health care to underserved population of remote areas in India is described. This paper presents the development and preliminary results of diagnostic validation of the application, intended for use among adult patients. The bilingual (English and Hindi) diagnostic tool consists of a core diagnostic section comprising a screening sub-module and criteria-based diagnostic sub-modules for 18 adult psychiatric disorders, and additional sections covering background information. The diagnostic tool of the application was examined among 100 consecutive consenting adult outpatients, by comparing it with detailed semi-structured clinical assessments led by a consultant psychiatrist, on accuracy of diagnoses generated, and examining the feasibility of its use.

**Results:**

The screening sub-module had high sensitivity and high specificity, low positive predictive values, but high negative predictive values for most disorders. For the diagnostic sub-modules, there was moderate (kappa = 0.4-0.6), to substantial agreement (kappa > 0.6) between diagnoses generated by the tool and consultants’ diagnoses, for all the disorders except dysthymia. Sensitivity was high barring a few disorders. Specificity was high for all the disorders, positive predictive values were acceptable to high for most disorders, and negative predictive values were consistently high. Completion rate was 100%; average time taken was five minutes for screening alone, and 30 minutes for complete assessment with screening and criteria-based evaluation. A majority of the patients, their relatives, and interviewers were satisfied with the interview.

**Conclusions:**

The preliminary results indicated that despite some limitations, the new diagnostic system was reasonably comprehensive, time-efficient and feasible, with an acceptable level of diagnostic accuracy. Hence, it appeared to be suitable for use as a telepsychiatric application.

## Background

Mental disorders are highly prevalent and equally disabling in India as in the rest of the world [1, 2], and as in other low-income countries, most people suffering from mental disorders continue to receive inadequate treatment [3]. Existing discrepancies between resources and need, urban and rural services, and primary, secondary and tertiary care, lead to what has been referred to as the ‘mental health gap’ [4]. Efforts are already underway in India to reduce the ‘mental health gap’ under the aegis of the National Mental Health Programme [5] by strengthening existing resources, developing new ones and enhancing manpower [4, 6]. However, these measures are highly resource intensive, and a long gestation period is expected before they begin to take effect. Telepsychiatry, the use of information and communication technologies to provide or support clinical psychiatric care from a distance, has been proposed as an alternative strategy [7]. Telepsychiatric programmes, which are being increasingly used in developed nations usually follow different approaches such as direct patient management though video-conferencing, consultation models, or collaborative-care models [8, 9]. However, in developing countries like India, such models are difficult to implement, because of resource and manpower constraints. Instead, a newer approach involving development of software packages with codified medical knowledge and logical decision support systems to aid assessment, diagnosis and management of psychiatric disorders could be a logical alternative [10]. With the broader aims of codifying medical knowledge, and providing an internet-based decision support system for diagnosis and treatment of psychiatric disorders, a pilot telepsychiatric project is underway at the department of psychiatry of the Postgraduate Institute of Medical Education and Research (PGIMER), Chandigarh, India. The department is the nodal centre of this service, with three peripheral sites located in the adjoining hill states of north-India. Right from its onset, this telepsychiatric project has followed a different model of training and enabling non-specialist (i.e., general physicians and para-professionals) personnel at remote sites to diagnose and treat mental illnesses on their own, with minimal consultation, supervision, or direct care from the nodal centre. To suit the needs of what has been construed by the authors as ‘tele-enabling’ model, in contrast to the existing ‘tele-consultation’ models, a computerised diagnostic and treatment application had to be constructed. For this purpose, a logically linked computerised decision support system for diagnosis and management of common psychiatric disorders in adults, and in children and adolescents, has been developed as a part of this project. This internet-based application is envisaged to enable non-specialists accessing it at remote areas to independently provide care. This requires 8 to 10 hours of training over two weeks, conducted through video-conferencing by the nodal centre. The software application has two separate decision support systems one for adults, and one for children and adolescents. In each case, the diagnostic system is logically linked to the pharmacological and psychological intervention modules taking into account the diagnosis, age, and medical illnesses. All the components of the decision system have been developed based on expert knowledge and guidelines, clinical experience and expertise; and utilize mainly ICD-10 [11] criteria.

For the diagnostic part of the application, the requirement was for a brief, but a comprehensive interview, which would generate reliable diagnoses and be simple to use. Two diagnostic interviews fulfilled these requirements; the well-validated Mini-International Neuropsychiatric Interview (MINI) [12], and a comparatively newer computerized clinical assessment tool, the Global Mental Health Assessment Tool – Primary Care Version (GMHAT-PC) [13]. Both the MINI [12] and the GMHAT-PC [13, 14] take about 15 to16 minutes for each assessment, while the time taken by other structured interviews ranges from 45 to 180 minutes [12]. However, because of copyright issues, none of these brief interviews could be used. Moreover, both instruments did not include many common disorders such as delirium, dementia, adjustment, somatoform and dissociative disorders, neurasthenia or sexual dysfunctions [12, 13]. Therefore, it was decided to develop an entirely new and indigenous diagnostic tool, in keeping with the aims of the telepsychiatry project described earlier.

This paper describes the development of a new diagnostic system for this net-based application. Apart from development, this initial pilot study also focuses on the accuracy of diagnoses generated by the tool, in comparison to more detailed semi-structured clinical assessments by teams led by consultant psychiatrists. Additionally, preliminary data on feasibility of use of this new diagnostic tool is also presented.

## Methods

### Basic approach and format of the diagnostic tool

The new net-based diagnostic tool was designed to consist of a comprehensive psychiatric clinical interview, albeit with a more focused and systematic approach. A balance between an objective criteria-based diagnostic exercise and conducting the interview in a conversational style was considered crucial, with the aim of replicating the routine clinical interview situation, where there is a greater need (as opposed to research settings) to establish a therapeutic relationship with the patient right from the onset of assessment. Moreover, the tool was primarily intended for use by non-specialists (i.e., general physicians and para-professionals) in remote- care settings. In keeping with these objectives, a structured format in simple language, easily understood by both patients and interviewers was adopted. It was decided that the diagnostic tool should cover both common and severe mental disorders. To meet the dual objectives of a comprehensive screening and a focused evaluation, a scheme of initial screening of patients for all disorders included in the tool, followed by a detailed assessment for specific disorders was incorporated. The interview was also segregated into ‘core’ and ‘additional’ sections to reduce the time required for the entire diagnostic interview to about half an hour. Diagnoses were based primarily on ICD 10 criteria, both from the Clinical Diagnostic Guidelines [11] and the Diagnostic Criteria for Research [15]. In certain parts (e.g. the diagnosis of delirium or dementia), DSM IV [16] criteria were used.

### The content and structure of the diagnostic tool

The diagnostic tool assesses for 18 disorders in adults, namely, delirium, dementia, mania (current and past), depression (current and past), dysthymia, psychosis, obsessive compulsive disorder, generalized anxiety disorder, panic disorder, phobias, reaction to severe stress and adjustment disorder, somatoform disorder, dissociative disorder, neurasthenia, sexual dysfunctions, alcohol dependence, substance dependence and mental retardation. It mainly assesses for current symptoms, except for symptoms of mood disorders, where both current and past symptoms are elicited. To avoid multiple diagnoses, it includes a diagnostic hierarchy by giving precedence to diagnostic categories appearing first in the ICD10 manuals.

**Figure 1:**
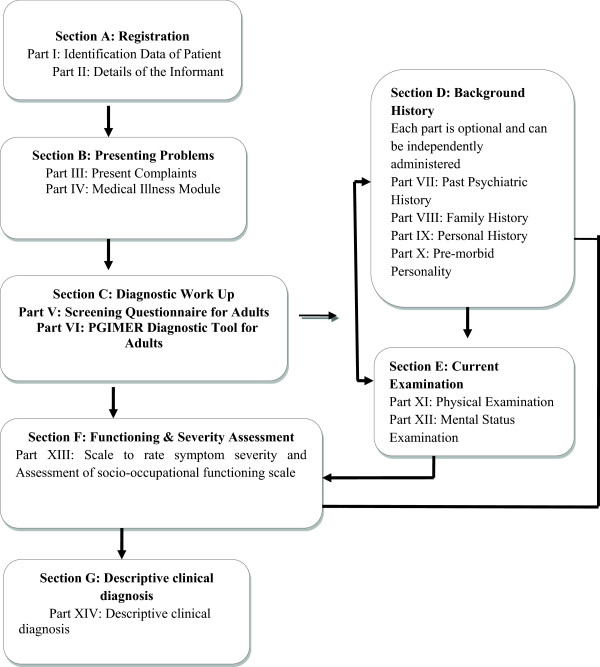
**Flow of the diagnostic tool.** The diagnostic exercise follows a stepwise approach to reach the final descriptive clinical diagnosis. First, the identification details, socio-demographic profile, presenting complaints and precipitating events are elicited and recorded (Sections A and B in the figure). This is followed by the ‘core’ diagnostic assessment (Section C in the figure), which includes initial screening for all disorders, followed by detailed criteria-based questions for specific disorders endorsed positive on screening. This ‘core’ diagnostic assessment is sufficient to generate a psychiatric diagnosis, but can be further supplemented by ‘additional’ sections on past, family, personal, developmental, medical and treatment history details (Section D), and physical and mental status examinations (Section E), whenever required. In addition, separate scales have been developed to assess symptom severity (on a five-point scale) and socio-occupational functioning (on a visual analogue scale) (Section F). At the end of the diagnpostic work up, a descriptive clinical diagnosis is generated (Section G).

The diagnostic exercise follows a stepwise approach to reach the final descriptive clinical diagnosis (see Figure 1). Firstly, the identification details, socio-demographic profile, presenting complaints and precipitating events are elicited and recorded. This is followed by the ‘core’ diagnostic assessment, which includes initial screening for all disorders, followed by detailed criteria-based questions for specific disorders endorsed positive on screening. The screening sub-module contains a total of 18 questions pertaining to the 18 disorders, and acts as the first gateway to the entire diagnostic exercise. Depending upon the positive responses on screening, the detailed diagnostic sub-modules open in an order based on an inbuilt hierarchy. In each diagnostic sub-module, there is a second-level enquiry about the primary or typical symptoms of that disorder, which proceeds with items pertaining to the other criteria only if the specified threshold is met at the second-level enquiry. Alternatively, it skips the remaining part of that sub-module and moves to the next sub-module as indicated in the first screening. This ‘core’ diagnostic assessment is sufficient to generate a psychiatric diagnosis, but can be further supplemented by ‘additional’ sections on past, family, personal, developmental, medical and treatment history details, and physical and mental status examinations, whenever required. In addition, separate scales have been developed to assess symptom severity (on a five-point scale) and socio-occupational functioning (on a visual analogue scale) at the end of diagnostic work-up.

**Figure 2:**
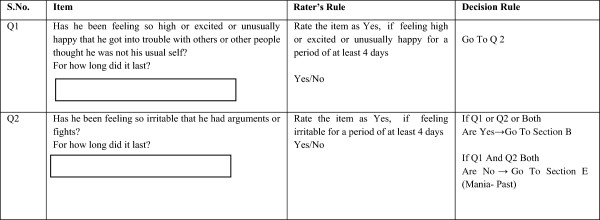
**Sample of the software requirement specifications to build the core diagnostic support system.** The core diagnostic support system contains diagnostic algorithms for the screening and diagnostic sub-modules. These algorithms consist of three main components, namely the question item with its serial number; the ‘rater’s rule’ for the rater to apply; and the ‘decision rule’ for computer automation. Each question item is based on the official classificatory systems, but is more descriptive, uses culturally relevant idioms and examples, and is simple to comprehend. For every question item in the screening and diagnostic sub-modules, a ‘rater’s rule’ has been framed in a ‘yes/no’ format. The rater’s rule specifies how the interviewer should rate an item as present or absent, based on the intent of the question, the duration and persistence of symptoms, and the distress or dysfunction caused by the symptoms. Thus, it incorporates a threshold for symptoms. The third component, the ‘decision rule’ is an automated rule that governs the flow of the diagnostic algorithm, as it defines how this ‘yes’ or ‘no’ response will influence the diagnostic decision tree. The ‘decision rules’ have been built based on the diagnostic thresholds set by standardized classification systems, as well as socio-cultural norms, duration of symptoms, possibility of self-limiting symptoms, and dysfunction caused by symptoms.

The diagnostic algorithm for the screening and diagnostic sub-modules consists of three main components, namely the question item with its serial number; the ‘rater’s rule’ for the rater to apply; and the ‘decision rule’ for computer automation (see Figure 2). Each question item is based on the official classificatory systems, but is more descriptive, uses culturally relevant idioms and examples, and is simple to comprehend. For every question item in the screening and diagnostic sub-modules, a ‘rater’s rule’ has been framed in a ‘yes/no’ format. The rater’s rule specifies how the interviewer should rate an item as present or absent, based on the intent of the question, the duration and persistence of symptoms, and the distress or dysfunction caused by the symptoms. Thus, it incorporates a threshold for symptoms. For example, in the sub-module for mania, the response to the question about ‘feeling high or unusually happy’ is considered positive only if the said behaviour ‘lasts for a period of at least 4 days’, as mentioned in the ‘rater’s rule’. There is also a provision for recording information, which is considered significant by the interviewer, but is not captured in the categorical ‘yes/no’ format. The third component, the ‘decision rule’ is an automated rule that governs the flow of the diagnostic algorithm, as it defines how this ‘yes’ or ‘no’ response will influence the diagnostic decision tree. The ‘decision rules’ have been built based on the diagnostic thresholds set by standardized classification systems, as well as socio-cultural norms, duration of symptoms, possibility of self-limiting symptoms, and dysfunction caused by symptoms. The ‘decision rules’ also incorporate hierarchical rules for skipping certain modules if a specific diagnosis is made, even though those modules are indicated by the screening (for example, if a diagnosis of schizophrenia is made, the module for anxiety disorders would not open, even if indicated by screening).

### Examining the diagnostic accuracy and feasibility of use of the diagnostic tool

The diagnostic accuracy and feasibility of the tool was examined for the English, as well as local language (Hindi) versions of the diagnostic tool as applicable to patients, at the department of psychiatry of PGIMER, the nodal centre, after receiving approval from the ‘Institution Ethics Committee’ of Post-Graduate Institute of Medical Education and Research (PGIMER), Chandigarh, India. For this purpose, consecutive outpatients aged more than 18 years, who gave written informed consent, were assessed using the new diagnostic tool. Interviews with the diagnostic tool were for the most part conducted by four psychologists who had Master’s level training in psychology, but had limited experience in diagnosis and assessment of patients. They were all trained by psychiatrists in the use of the diagnostic tool. Patients were randomly allocated to undertake the tool-based assessment followed by consultant’s evaluation, or vice versa. Clinical assessments were undertaken in the department by team of a trainee psychiatrist and a consultant psychiatrist. These detailed semi-structured assessments also used ICD10 diagnostic criteria and generated ICD10 diagnoses. The interviewers using the new diagnostic tool were blind to the consultant’s diagnosis, and the consultant psychiatrist was blind to the diagnostic tool based assessment. Demographic data, ratings on screening and diagnostic modules, diagnoses generated by the tool and the consultants’ diagnoses were all recorded. Additionally, details regarding the completion rate, the total time taken, and feedback on comprehensibility of language, style of questioning and satisfaction, were also obtained from interviewers, the patients and the relatives accompanying them. The required sample size for kappa values of 0.4 to 0.6, at a power of 80% and alpha of 0.05, was estimated to be 100.

### Data analysis

Statistical analysis was carried out using the Statistical Package for Social Sciences-version fifteen [17]. Apart from descriptive analyses, paired t-tests were conducted to compare the mean number of diagnosis generated by the two interviews. Analyses were carried out both for the broad ICD 10 diagnostic categories, as well as some individual categories belonging to mood and neurotic disorders. Sensitivity, specificity, positive and negative predictive values were computed for the screening and the diagnostic sub-modules of the tool, compared to the consultant’s diagnosis. Additionally, kappa coefficients were computed to assess agreement between the diagnoses generated by the diagnostic sub-modules and the consultant’s diagnosis.

## Results

Over a period of 6 months a total of 107 persons were included in the study; seven of these had to be excluded, because a clear diagnosis could not be made after the consultant’s evaluation. About half of these patients (n = 54) were assessed on the diagnostic tool first, while the remaining patients underwent consultants’ evaluation prior to assessment with the tool.

### Demographic profile of participants

Table 1 depicts the demographic profile of the 100 patients included in the study.

**Table 1:** **Demographic details of participants (N = 100**)

Variables		Mean (SD)	Frequency (%)
**Age (In years)**		35.24 (13.32)	
**Years of education**		9.73 (4.44)	
**Gender**	Male		53 (53%)
	Female		47 (47%)
**Marital status**	Married		69 (69%)
	Single		26 (26%)
	Others		5 (5%)
**Occupation**	Employed		39 (39%)
	Unemployed		9 (9%)
	Housewife/engaged in household work		36 (36%)
	Retired		2 (2%)
	Student		14 (14%)
**Family Type**	Nuclear		47 (47%)
	Joint		53 (53%)
**Locality**	Rural		43 (43%)
	Urban and semi-urban		57 (57%)

Patients were middle-aged, with more men than women constituting the study sample. They were mostly educated, married, either employed or engaged in household work, and mainly came from urban or semi-urban areas. This corresponded to the usual profile of attendees at this centre.

### Diagnostic profile of participants

The mean number of total diagnoses made by the diagnostic sub-modules of the tool was 1.38 (SD 0.82), and that made by the consultant’s evaluation was 1.23 (SD 0.48). No significant difference was found between the mean numbers of diagnoses between the two methods of assessment. Organic disorders were diagnosed in 2% of the patients, alcohol use disorder in 4%, substance use disorders in 6%, psychoses in 18%, mood disorders (bipolar disorder, major depression, dysthymia) in 47%, neurotic, stress related and somatoform disorders in 40%, disorders of sexual functioning in 1%, and mental retardation in 5% by the diagnostic tool. No diagnosis could be reached by the tool in four patients, and consultants’ diagnoses for these patients were somatoform disorder, anxiety disorder, organic mood disorder and psychosis respectively. The frequencies of diagnoses in each category made by the diagnostic tool and by the consultant’s evaluation are included in Table 2.

**Table 2:** **Frequency of diagnoses and agreement between diagnoses made by diagnostic sub-module and the clinical interview**

	Diagnostic tool	Clinical interview							
Disorders	Frequency n (%)	Frequency n (%)	T P	FP	Cohen’s kappa ( ***k***)	Sensitivity	Specificity	Positive predictive value	Negative predictive value
			**FN**	**TN**					
Organic mental disorders	2 (2%)	5 (5%)	2	0	0.56	0.4	1	1	0.97
			3	95					
Alcohol dependence syndrome	4 (4%)	3 (3%)	3	1	0.85	1	0.99	0.75	1
			0	93					
Drug dependence syndrome	6 (6%)	4 (4%)	3	3	0.58	0.75	0.97	0.5	0.99
			1	93					
Psychotic disorders	18 (18%)	16 (16%)	13	5	0.72	0.81	0.94	0.72	0.96
			3	79					
Any Mood Disorder	47 (47%)	44 (44%)	36	11	0.62	0.82	0.80	0.77	0.85
			8	45					
Bipolar affective disorder	15 (15%)	14 (14%)	10	5	0.64	0.71	0.94	0.67	0.95
			4	81					
Manic episode	11 (11%)	7 (7%)	6	5	0.64	0.86	0.95	0.55	0.99
			1	88					
Depressive episode/Recurrent depressive disorder	29 (29%)	25 (25%)	18	11	0.54	0.72	0.85	0.62	0.90
			7	64					
Dysthymia	6 (6%)	5 (5%)	1	5	0.14	0.2	0.95	0.16	0.96
			4	90					
Any Neurotic, Stress related & Somatoform disorders	40 (40%)	39 (39%)	31	9	0.64	0.80	0.85	0.78	0.87
			8	52					
Anxiety (GAD, panic disorder, phobias) and stress related disorders	17 (17%)	16 (16%)	9	8	0.46	0.56	0.90	0.53	0.92
			7	76					
OCD	11 (11%)	9 (9%)	9	2	0.89	1	0.98	0.82	1
			0	89					
Somatoform Disorders	17 (17%)	9 (9%)	8	9	0.56	0.89	0.90	0.47	0.99
			1	82					
Dissociative disorders	5 (5%)	6 (6%)	3	2	0.52	0.50	0.98	0.6	0.97
			3	92					
Sexual dysfunction	1 (1%)	2 (2%)	1	0	0.66	0.5	1	1	0.99
			1	98					
Mental Retardation	5 (5%)	7 (7%)	5	0	0.82	0.71	1	1	0.98
			2	93					

### Agreement between the diagnostic tool and consultants’ diagnoses

#### *a.*Screening sub-module of the diagnostic tool

Table 3 depicts the sensitivity, specificity, positive predictive value and negative predictive values of the screening sub-module of the diagnostic tool, compared to the diagnoses obtained following the consultant’s evaluation.

**Table 3:** **Sensitivity, specificity, positive and negative predictive values of screening sub-module of the diagnostic tool**

			Clinical interview					
			+	-				
Disorders	Screening of diagnostic tool	+	T P	FP	Sensitivity	Specificity	Positive predictive value	Negative predictive value
		-	FN	TN				
Organic mental disorders			3	3	0.6	0.97	0.5	0.98
			2	92				
Alcohol dependence syndrome			3	4	1	0.96	0.43	1
			0	93				
Drug dependence syndrome			4	7	1	0.93	0.36	1
			0	89				
Psychotic disorders			12	19	0.75	0.77	0.39	0.94
			4	65				
Any Mood Disorder			40	29	0.90	0.48	0.58	0.87
			4	27				
Any Neurotic, Stress related & Somatoform disorders			36	44	0.92	0.28	0.45	0.85
			3	17				
Anxiety (generalized anxiety, panic and phobic disorder) and Stress-related disorders			14	55	0.87	0.35	0.20	0.94
			2	29				
OCD			7	18	0.78	0.80	0.28	0.97
			2	73				
Somatoform Disorders			8	25	0.89	0.73	0.24	0.96
			1	66				
Dissociative disorders			4	5	0.67	0.95	0.44	0.98
			2	89				
Sexual dysfunctions			1	4	0.5	0.96	0.2	0.99
			1	94				
Mental retardation			6	4	0.86	0.96	0.6	0.99
			1	89				

The results show that the sensitivity of screening part of the tool was high for most disorders except organic brain disorders (60%) and sexual dysfunctions (50%). This was because of the small number of patients in these categories, and the high false negative rates obtained in both these categories. The specificity of screening was also high for most of the disorders, apart from the broad category of mood disorders (48%) and the category of neurotic and stress-related disorders (28%), where it was mainly compromised by low specificity of the diagnoses of generalized anxiety, panic, phobic and stress-related disorders. In all these categories, low specificity appeared to result from the relatively low rates of true negative cases. Positive predictive values were low for almost all disorders, except mood disorder and mental retardation. This was a reflection of the high rates of false positive diagnoses in most categories. On the other hand, negative predictive values were consistently high because of the high rates of true negative cases in most categories.

#### b. Diagnostic sub-modules of the diagnostic tool

Table 2 depicts the Cohen’s kappa values, sensitivity, specificity, and positive and negative predictive values of the diagnostic sub-modules of the tool, compared to consultants’ diagnoses.

Cohen’ kappa values revealed substantial (>0.6) to near perfect agreement for alcohol dependence, psychosis, mood disorders (broad category), bipolar disorder, hypomania/mania (current), unipolar and bipolar depression (current), neurotic, stress related and somatoform disorders, obsessive compulsive disorder, sexual dysfunction and mental retardation. There moderate agreement (0.4 - 0.6) for organic disorders, drug dependence, anxiety and stress-related disorders, and somatoform and dissociative disorders. Low agreement was seen for dysthymia (0.14) alone. Sensitivity of diagnoses was high, apart from dysthymia (20%), organic disorders (40%), generalized anxiety, panic, phobic, and stress-related disorders (46%), sexual dysfunctions (50%) dissociative disorders (52%) and somatoform disorders (54%). Low sensitivities were primarily due to the high rates of false negative cases. Unlike the screening section of the tool, specificity was high for all the disorders, and positive predictive values were acceptable to high for most disorders, except dysthymia (0.16) and dissociative disorders (0.47). Thus, the numbers of false positive diagnoses were reduced after applying the diagnostic sub-modules. Similar to the screening sub-module, negative predictive values were consistently, high because of the high rates of true negative cases.

#### c. Discordance analysis

The number of discordant cases in each diagnostic category is shown in Table 4. The maximum number of discordant cases was seen in mood disorders, and neurotic, stress -related and somatoform disorders. Most of discordance between tool-based and clinical diagnoses was accounted for by the two broad categories of unipolar and bipolar depression (current), and anxiety (generalized anxiety disorder, panic disorder, phobias) and stress-related disorders. Eight discordant cases in the category of current depression were diagnosed by consultants as having dysthymia or a neurotic disorder. Eleven out of 15 discordant cases in the category of anxiety (generalized anxiety disorder, panic disorder, phobias) and stress related disorders were diagnosed by consultants as having another neurotic and stress-related disorder or depression.

**Table 4:** **Frequency of concordant and discordant cases in each diagnostic category**

Disorders	Number of cases where agreement occurred n (%)	Number of cases where disagreement occurred n (%)
Organic mental disorders	95 (95)	5 (5)
Alcohol dependence syndrome	99 (99)	1 (1)
Drug dependence syndrome	96 (96)	4 (4)
Psychotic disorders	92 (92)	8 (8)
Any Mood Disorder	77 (77)	23 (23)
BPAD	88 (88)	12 (12)
Depressive episode	83 (83)	17 (17)
Dysthymia	90 (90)	10 (10)
Any Neurotic, Stress related & Somatoform disorders	81 (81)	19 (19)
Anxiety (GAD, panic disorder, phobias) and stress related disorders	74 (74)	26 (26)
OCD	98 (98)	2 (2)
Somatoform Disorders	90 (90)	10 (10)
Dissociative disorders	96 (96)	4 (4)
Sexual dysfunction	99 (99)	1 (1)
Mental Retardation	98 (98)	2 (2)

### Duration of assessment using the new diagnostic tool

Details regarding the duration of assessment by the new tool are shown in Table 5. The time taken for assessment by the ‘core’ diagnostic tool was about 30 minutes, and that for screening alone was about five minutes.

**Table 5:** **Results of the feasibility analysis of the diagnostic tool**

			Interviewee/informants	Interviewer
			Frequency N = 100	Frequency N = 100
**Time taken for diagnostic assessment**	Mean - 30.69 (SD 10.87) minutes; Median - 29.5 minutes; Range: 10 – 60 minutes			
**Time taken for screening alone**	Mean - 4.85 (SD 0.86) minutes; Median - 5 minutes; Range: 3 – 8 minutes			
**Completion rate**	100%			
**Overall satisfaction**		Very dissatisfied	0	0
		Dissatisfied	9	14
		Satisfied	57	66
		Very satisfied	34	20
**Comprehensibility of language**		Very difficult	0	0
		Difficult	9	11
		Easy	70	65
		Very easy	21	24
**Style of questioning**		Very difficult	1	6
		Difficult	13	17
		Easy	62	54
		Very easy	24	23
**Extent to which presenting complaints were addressed**		Very dissatisfied	2	4
		Dissatisfied	8	10
		Satisfied	65	58
		Very satisfied	25	28

### Feasibility of use of the new diagnostic tool

Results regarding feasibility of use are also are depicted in Table 5.

All the participants completed the entire interview. A majority of the patients, their relatives and the persons conducting the assessment were satisfied with the interview, especially with the language used and the style of questioning, as well the extent to which the presenting complaints were addressed by the interview.

## Discussion

Assessing and detecting psychiatric disorders in general health care settings, particularly in developing countries like India, is a highly challenging task [18]. Several strategies including training of general health workers or physicians have been proposed [18], and tried in the National Mental Health Programme [5], but these strategies have numerous problems such as inadequate training, short-lived gains of the limited training received, lack of motivation of personnel, and lack of support by psychiatrists. It is, therefore, necessary to look for additional alternatives, which do not rely on presence of psychiatrist for every patient, and yet can deliver specialized care in the most effective manner. The net-based tool for diagnosing and treating psychiatric disorders in adults developed as part of this project is envisaged to fulfil many of these needs. The advantages of the tool are that: it is based on standardized classificatory systems; it is structured, fully automated, with an in-built logical support system for diagnosis management: it has facilities for real-time and post-interview monitoring and support, whenever required. These features not only enhance the performance of the tool, but also empower the interviewer to carry out the process of diagnosis satisfactorily.

The primary focus of the current paper was to describe the development of the tool, and present the preliminary findings regarding the performance of the tool as a screening and a diagnostic instrument, which would justify its use as a part of the telepsychiatric application. Existing validated interviews such as the MINI could not be used, mainly because of issues regarding copyright. Therefore, an entirely new diagnostic tool had to be constructed. Being a pilot and proof-of-concept study of this new diagnostic tool, certain limitations were inherent in its design. Firstly, though the sample size was adequate based on targeted kappa values, a much larger sample would eventually be required to test the diagnostic accuracy of the entire tool. The numbers involved for many diagnostic categories were small. Therefore, the agreement between diagnoses generated by the tool and consultants’ diagnoses were tested for the broad categories in case of neurotic disorders, and not individual disorders. Secondly, the comparison was carried out with detailed semi-structured assessments made by a team headed by a consultant psychiatrist. Ideally, another structured interview could have been used for comparison, as it has been noted that reliability of open-form clinical interviews is comparatively low [19], though it can be improved by using diagnostic criteria [20], as is the case in our clinical setting. Other studies have also relied on consultants’ diagnoses in the absence of a structured interview comparison, or even otherwise [21, 22]. Finally, at this initial stage only a pre-computerised version of the diagnostic tool could be tested. The aim was again to gather as much data and experience with what was an entirely new diagnostic interview, before going in for online activation and testing. The results of this study need to be viewed in the light of these limitations.

Screening instruments for psychiatric disorders should include commonly encountered disorders and have high sensitivity for detection of these disorders, owing to their disabling consequences [23]. The screening sub-module of the diagnostic tool fulfilled both these criteria, as it included a broad range of commonly prevalent disorders and, with certain exceptions, had a high sensitivity for detecting these disorders. Sensitivity was low for organic disorders, because some of these were labelled as ‘functional’ psychiatric disorders, a fallacy that has been commonly noted earlier [24]. Low sensitivity in case of sexual dysfunctions was due to the inability to detect one patient, presumably because of the sensitive nature of the subject [25]. The specificity of screening was high for most disorders as well, except for the broad category of mood disorders and neurotic and stress-related disorders, where it was mainly compromised by low specificity of the diagnoses of generalized anxiety, panic, phobic and stress-related disorders. Low specificity of screening instruments for the milder psychiatric disorders, particularly anxiety disorders, is common with other screening tools as well [26]. Positive predictive values were also low for most disorders, which indicate a high rate of false positive diagnoses. This is another problem that has been noted for psychiatric screening tools [27, 28]. However, a substantial number of patients with false positive results meet diagnostic criteria for other mental disorders, and the burden of follow-up assessments for patients with positive screens is usually not too high. Hence, it has been suggested that erring on the side of sensitivity is preferable for instruments screening for psychiatric disorders [29]. Finally, the consistently high negative predictive values indicated that the screening module was correctly identifying all those without a psychiatric disorder who would not require any further psychiatric intervention.

Although the mean number of diagnoses generated by the diagnostic sub-modules of the tool was somewhat higher than the clinical evaluation, this difference was not significant. This finding was unlike that seen with structured interviews; for example, the Composite International Diagnostic Interview-Auto (CIDI-Auto) generates a mean number of 2.53 diagnoses per patient [30]. While this may be useful in research interviews, it might present problems for routine clinical evaluation. In this regard, the inbuilt hierarchies and exclusion rules of the diagnostic tool reduced the chances of multiple diagnoses, thereby simplifying the entire process for the interviewer.

The kappa values indicated moderate (0.4-0.6) to high (>0.6) agreement between the two assessments for all disorders, except dysthymia. Moreover, sensitivity of the detailed diagnostic sub-modules was high apart from dysthymia, organic disorders, generalized anxiety, panic, phobic and stress-related disorders, sexual dysfunctions, and dissociative and somatoform disorders. Unlike the screening module, specificity was high for all the disorders and positive predictive values were acceptable to high for most disorders, except dysthymia and dissociative disorders, while negative predictive values continued to remain high for all disorders. Discordance analysis revealed that for common mental disorders including depression, dysthymia, and anxiety and stress-related disorders, a large number of cases were classified interchangeably, which might explain the comparatively low level of agreement seen with these disorders. Overall, considering the kappa values, sensitivity and the proportion of discordant diagnoses, the diagnostic tool performed relatively poorly for the minor or common mental disorders. Similarly, with other diagnostic interviews such as the NIMH-Diagnostic Interview Schedule (DIS), the CIDI, or the MINI, agreement for dysthymia, anxiety disorders and somatoform disorders has usually been found to be lower when compared to clinical diagnosis, as clinicians regarded other diagnoses as dominant, and did not diagnose anxiety disorders separately though they agreed on the presence of symptoms [12, 22, 31]. Nevertheless, these results suggested that refinement of diagnostic criteria were needed to improve this aspect of the diagnostic tool.

Finally, the feasibility analysis demonstrated that the instrument was easy to administer and was rated highly both by the interviewer and the interviewee. In the new tool a lot of emphasis has been placed on flexibility of interviewing and establishing a therapeutic relationship with the patient, without compromising on the objectivity of assessment. The feasibility analysis suggested that these features of the tool helped it recreate the clinical situation to a large extent. All this was achieved in a time of about 30 minutes, which was about a-third of that required for detailed clinical evaluations. Only the MINI [12] and the GMHAT/PC [14] take less time. Moreover, the time taken for using the screening sub-module was about five minutes, which was in keeping with the suggestion that screening time should not be more than about five minutes if an instrument is to be widely adopted [12].

Therefore, despite some methodological limitations and concerns about certain parts of the new diagnostic tool, the findings of this preliminary study suggested that it could prove suitable for use as a part of an online telepsychiatric application. Efforts are already underway to further enhance the accuracy of the online version of the diagnostic tool, to test its validity at the primary and secondary care levels with larger samples, and to validate it against other structured interviews such as the MINI.

## Conclusions

The development of a fully structured and automated diagnostic system for adult psychiatric disorders is described. Preliminary results suggested that despite some limitations, diagnoses generated by the tool had an acceptable level of accuracy, and the tool appeared to be feasible to use. Therefore, it seemed to be suitable for use as a part of an online telepsychiatric application meant for diagnosis and management of adult psychiatric disorders.

## Abbreviations

ICD 10: International Classification of Diseases – Tenth Revision
DSM IV: Diagnostic and Statistical Manual of Mental Disorders – Fourth Edition
SCID: Structured Clinical Interview for DSM IV disorders
MINI: Mini-International Neuropsychiatric Interview
CIDI: Composite International Diagnostic Interview
GMHAT-PC: Global Mental Health Assessment Tool – Primary Care Version
DIS: The NIMH-Diagnostic Interview Schedule.
